# Drift and dispersion of silver carp (*Hypophthalmichthys molitrix*) eggs and larvae for hypothetical spawning scenarios in the Upper Mississippi River

**DOI:** 10.1038/s41598-026-41803-w

**Published:** 2026-05-06

**Authors:** Jessica Z. LeRoy, Grace L. Loppnow, P. Ryan Jackson, G. Everett Lasher

**Affiliations:** 1 U.S. Geological Survey, Central Midwest Water Science Center, Urbana, IL 61801 USA; 2 Minnesota Department of Natural Resources, Saint Paul, MN 55155 USA

**Keywords:** Invasive carp, silver carp, spawning, drift modeling, Upper Mississippi River, invasive species, Hydrology, Invasive species

## Abstract

Invasive carp pose ecological and economic risks to North American freshwater systems. This study uses the Fluvial Egg Drift Simulator to model the drift of invasive silver carp (*Hypophthalmichthys molitrix)* eggs and larvae after hypothetical spawning in Pools 1–10 of the Upper Mississippi River. Although adult invasive carps have been detected in this region, no reproduction has been confirmed as of this publication. A total of 450 spawning scenarios were simulated, representing 5 water temperatures, 9 flows, and 10 spawning locations in the tailwaters of lock and dam structures. The study examined egg and larval positions at two key developmental stages: hatching and gas bladder inflation, when larvae seek nursery habitat. Under a wide variety of flow conditions and water temperatures, eggs spawned upstream from Lake Pepin (Pool 4) are likely to settle in the lake before hatching, possibly increasing mortality rates. Eggs that survive passage through Lake Pepin reach gas bladder inflation within the study area, except in scenarios with lower temperatures and higher flows. Conversely, larvae spawned downstream from Lake Pepin generally drift out of the study area before reaching gas bladder inflation, except in cases of higher temperatures and lower flows. These findings inform ichthyoplankton sampling strategies and management actions aimed at reducing invasive carp populations in areas likely to support recruitment.

**Supplementary Information:**

The online version contains supplementary material available at 10.1038/s41598-026-41803-w.

## Introduction

Four invasive carp species threaten freshwater ecosystems as well as economic and recreational uses of North American waters – silver carp (*Hypophthalmichthys molitrix*), bighead carp (*H. nobilis*), grass carp (*Ctenopharyngodon idella*), and black carp (*Mylopharyngodon piceus*). Since their introduction in the 1960s and 1970s, invasive carps have spread throughout much of the Mississippi River Basin, including into the Upper Mississippi River (UMR) and some of its tributaries^1,2^. Bighead and silver carp are the most abundant of the four species in the Mississippi River Basin^2,3^. Established populations of bighead and silver carp are present in the UMR below Lock and Dam (LD) 19, where reproduction and recruitment have been documented^4,5^. Bighead and silver carp eggs and larvae have been collected as far upstream as Pool 16, and adults have been detected as far upstream as Pool 2 near Hastings, Minnesota (MN) and in the lower St. Croix and Minnesota Rivers^4–6^. Grass carp have been detected throughout the UMR^7^, and black carp have been detected below LD 19^3,4^.

The four invasive carp species are broadcast spawners with high fecundity and share similar spawning cues and behaviors^1,8^. Spawning typically occurs in the spring or summer, when high or rising water levels^1^ coincide with water temperatures ranging from about 18 to 27 degrees Celsius (°C)^9–18^. Invasive carp seek out fast, turbulent flows for spawning, which can naturally occur at confluences, channel constrictions, rapids/hardpoints, or islands^1,14,19–22^, or at human-made structures such as dams^21,23^. The velocity conditions that are suitable for invasive carp spawning may span from 0.5 to 2.5 meters per second (m/s)^23,24^, though a narrower range of 1.0 to 1.6 m/s was observed in laboratory experiments for silver carp^18^. The same laboratory study also showed that fertilization of silver carp eggs was maximized at 1.2 to 1.4 m/s^18^.

The development rate of the early life stages of bighead, silver, and grass carp is temperature and species dependent^25–28^. Once fertilized, invasive carp eggs undergo a period of water hardening in which they absorb water, increase in size, and decrease in density over a period of about 1.5 to 3 hours (h)^25–27^. The water-hardened eggs continue to drift until hatching, generally about 17–19 hours post fertilization (hpf) at 28 °C and 53–60 hpf at 18 °C^25–27^. Upon hatching, the larvae can swim vertically^25,27,29,30^ but continue to drift with the flow of the river. The larvae reach the gas bladder inflation stage (GBI, or developmental stage 38) at 74–80 hpf at 28 °C and 231–252 hpf at 18 °C^25–27^. After reaching the GBI stage, the larvae can swim laterally and begin to seek out nursery habitat and food, typically in backwater and side channel environments^1,28^.

Several authors note the importance of the period of drift for successful invasive carp recruitment^1,10,19,31–33^. Many riverine fish have a drift phase in their development, which affords a number of advantages^28,34^. Flowing water is typically well-mixed and well-oxygenated, and predation can be limited by turbidity as well as the effort required to feed on broadly distributed eggs/larvae^28^. Eggs that settle to the bed may be damaged by abrasion or impacts^30,35^ or suffocate due to burial in sediment^1,36^, though the extent to which settling affects mortality rates in known spawning rivers is not clear. Although invasive carp eggs are nearly neutrally buoyant once water-hardened, some turbulence is required to keep eggs in suspension^25–28^. However, egg mortality rates have been shown to increase with increasing turbulent kinetic energy in a laboratory turbulence tank^35^.

As detections and captures of invasive carp in the UMR have increased, the UMR has become the focus of intensive monitoring, management, and research coordinated through the Mississippi Interstate Cooperative Resource Association^2,37,38^. Comprehensive monitoring of all life stages of invasive carp is a key management strategy to inform decision-making^37^. Sampling for early life stages (i.e., eggs and larvae) and understanding egg and larval drift is essential for determining where the invasion front may be advancing and where response actions may be needed. Crews typically sample the UMR for invasive carp eggs and larvae in spring and summer, when the water temperatures are in the observed range for spawning^5,38,39^. A better understanding of which pools are suitable for spawning and recruitment would allow managers to target sampling and management actions where they may be most effective, which is critical when resources for monitoring and response are limited.

The suitability of a river, reach, or pool for invasive carp spawning depends on several factors, including accessibility to adult fish, availability of spawning sites, hydraulic and water-quality characteristics that promote egg survival, and availability of nursery habitat and food for exogenously feeding larvae (post-GBI) and juvenile fish^40^. The Fluvial Egg Drift Simulator (FluEgg) was developed by the U.S. Geological Survey to evaluate the suitability of rivers for invasive carp spawning by simulating the drift and biological development of invasive carp eggs and larvae from fertilization until the GBI stage^41–45^. FluEgg simulations, which track the three-dimensional (3D) position of individual eggs and larvae as they drift downstream in a simplified rectangular prismatic representation of the river channel, can be used to evaluate the invasive carp spawning suitability based on the assumption that survival and recruitment are linked to (1) eggs remaining in suspension until hatching^27,28,36,41^, and (2) larvae reaching the GBI stage near nursery habitat^40^. In addition to spawning suitability analyses^40,41^, FluEgg has been used to identify probable spawning areas of sampled eggs/larvae^21,23,46^, analyze egg age distributions in field samples^47^, and to characterize the optimal conditions for egg/larval drift in rivers with known invasive carp spawning^48^.

In this study, we applied FluEgg to examine *hypothetical* silver carp spawning and egg/larval drift in Pools 1–10 of the UMR for all combinations of 5 water temperatures, 9 flows, and 10 spawning locations (in Pools 1–9) for a total of 450 scenarios. The scenarios were selected based on the current (2025) understanding of invasive carp spawning requirements as no invasive carp reproduction has been observed or documented in this reach of the UMR at the time of this publication. The spawning locations were placed in the tailwaters of the lock and dams in this reach, where flows are typically fast and turbulent and where invasive carp migrating upstream may encounter a barrier to further movement. The hydraulic data needed to run FluEgg were supplied by an existing unsteady (time-varying) hydraulic model^49^. Instantaneous hydraulic profiles were extracted from the hydraulic model and used as an approximation of steady (unchanging in time) flows. We used the results of the FluEgg simulations to compare where eggs hatch and larvae reach the GBI stage among the scenarios. We also identified the scenarios in which the eggs are likely to remain in suspension until hatching. These results were used to assess the relative risk posed by the hypothetical spawning scenarios for invasive carp recruitment in Pools 1–10 of the UMR.

## Methods

The drift of silver carp eggs and larvae in the UMR was simulated using FluEgg version 4.1.1^44^ for 450 hypothetical spawning scenarios over a wide range of flows (Table 1). The hydraulic inputs for these FluEgg simulations were obtained from an existing one-dimensional (1D) Hydrologic Engineering Center River Analysis System (HEC-RAS) version 5.0.7 model^49^. The inputs and outputs for the FluEgg simulations are archived and publicly available^50^. The study area, hydraulic inputs, and FluEgg simulations are described below.

**Table 1:** Nominal flows used for hypothetical spawning scenarios in this study, specified as the flow at U.S. Geological Survey streamgage Mississippi River at Winona, MN (05378500). (m^3^/s: cubic meters per second, cfs: cubic feet per second)

Flow (m^3^/s)	Flow (cfs)
850	30,000
1,130	40,000
1,560	55,000
2,270	80,000
2,830	100,000
3,400	120,000
3,960	140,000
4,530	160,000
5,100	180,000

### Study area description

**Fig. 1:**
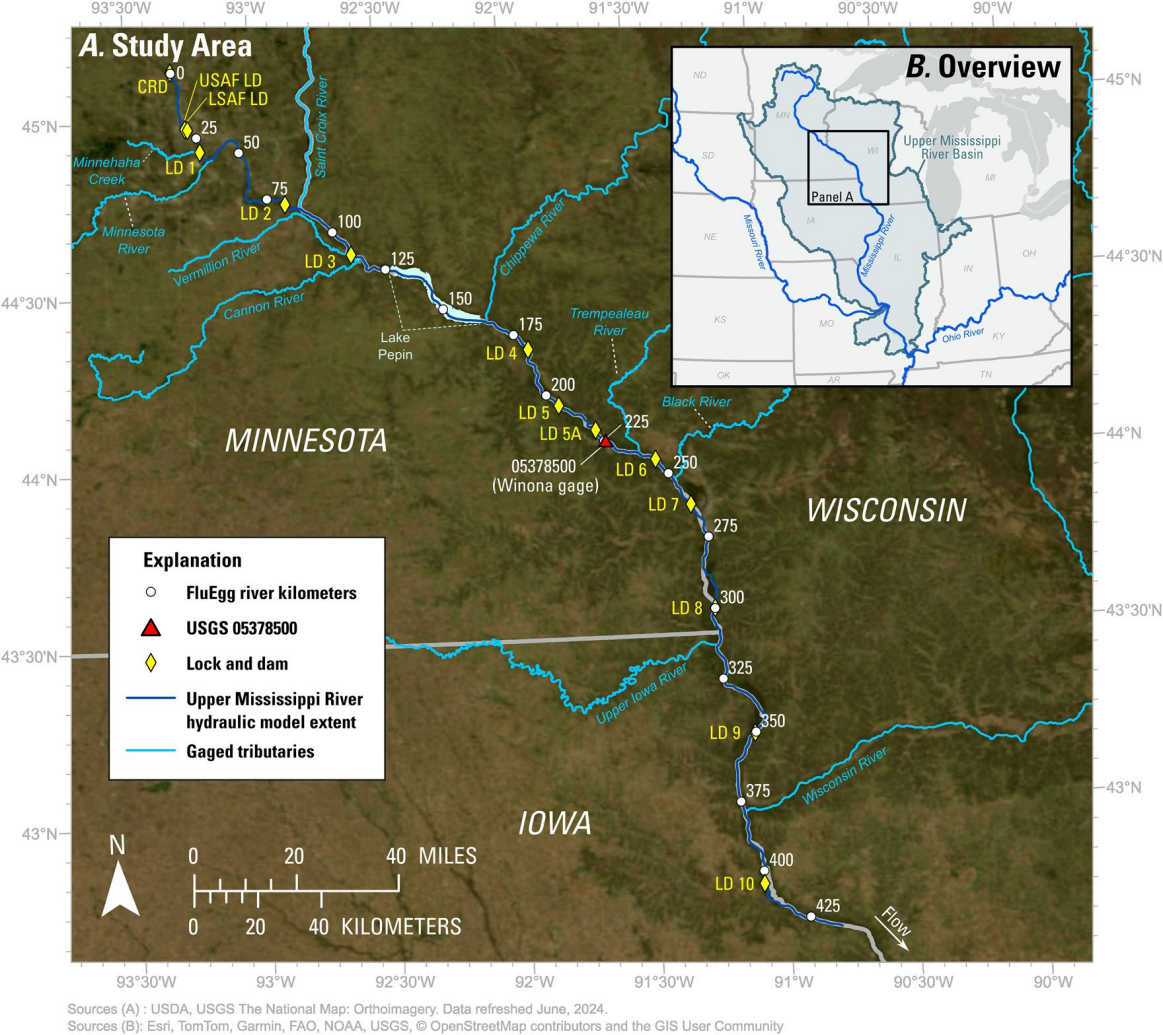
(**A**) Map of the Upper Mississippi River study area showing U.S. Geological Survey (USGS) streamgage Mississippi River at Winona, MN (05378500), locks and dams (LD), gaged tributaries, and the river kilometer markers for the coordinate system used in FluEgg. (**B**) Overview map showing regional context of the study area. Maps were created with ESRI ArcGIS Pro 3.5.5 (https://www.esri.com/en-us/arcgis/products/arcgis-pro/overview). Satellite basemap imagery sources: U.S. Department of Agriculture, USGS The National Map.

The UMR is a 2,100 km [km] waterway that spans five U.S. states – Minnesota, Wisconsin, Iowa, Illinois, and Missouri – from its headwaters in Minnesota to its confluence with the Ohio River (not shown) (Fig. 1). The UMR and adjacent terrestrial habitats are home to a wide variety of plants, fish, and wildlife, including 39 rare, endangered, or threatened fish species^51^. The UMR also supports a variety of recreational and commercial uses, such as fishing and navigation. Navigation along the UMR is facilitated by 29 locks and dams, built between 1895 and 1968, as well as dredging and river training structures that maintain a 9-foot deep navigation channel^52,53^. The lock at Upper St. Anthony Falls (USAF LD) on the UMR has been permanently closed since June 2015 to prevent the upstream movement of invasive carps^38,54^. Below St. Anthony Falls, the gates of each dam are adjustable from fully closed to fully open (open-river conditions)^52,53^.

This study focuses on Pools 1–10 of the upper impounded reach of the UMR, from Coon Rapids Dam (CRD) to about 29.8 km downstream from LD 10 (Fig. 1). Locations in the study area are referenced by river kilometers (rkm) that increase with distance downstream from Coon Rapids Dam at 0 rkm. This reference system differs from the standard river miles (rm) used by the U.S. Army Corps of Engineers (USACE), which decrease with distance downstream from Coon Rapids Dam at rm 866. Throughout most of the study area, the 9-foot navigation channel is accompanied by a complex assemblage of side channels, islands, and backwater lakes. Lake Pepin, in Pool 4, is unique among the lakes along this reach in that it is a natural lake formed behind the sediment deposited at the mouth of the Chippewa River^55^ and has since served as a trap for sediments arriving from upstream^56^. There are relatively few levees in the upper impounded reach of the UMR, thus the floodplain is still hydrologically connected to the river^51^. Major tributaries in the study area include the Minnesota, St. Croix, Chippewa, and Wisconsin Rivers.

### Selection of hypothetical spawning scenarios

Although adult silver carp, grass carp, and bighead carp have been detected and captured in the study reach^6,57^, no evidence of reproduction has yet been confirmed in Pools 1–10 of the UMR at the time of this publication. Therefore, *hypothetical* spawning scenarios in the UMR were devised for this study based on current understanding of invasive carp spawning behaviors. The hypothetical scenarios comprise all combinations of 5 water temperatures, 9 flows (Table 1), and 10 spawning locations for a total of 450 scenarios. The 5 water temperatures (18, 20, 22, 24, and 26 °C) span most of the typical range (18 to 27 °C) during invasive carp spawning^9–18^.

**Fig. 2:**
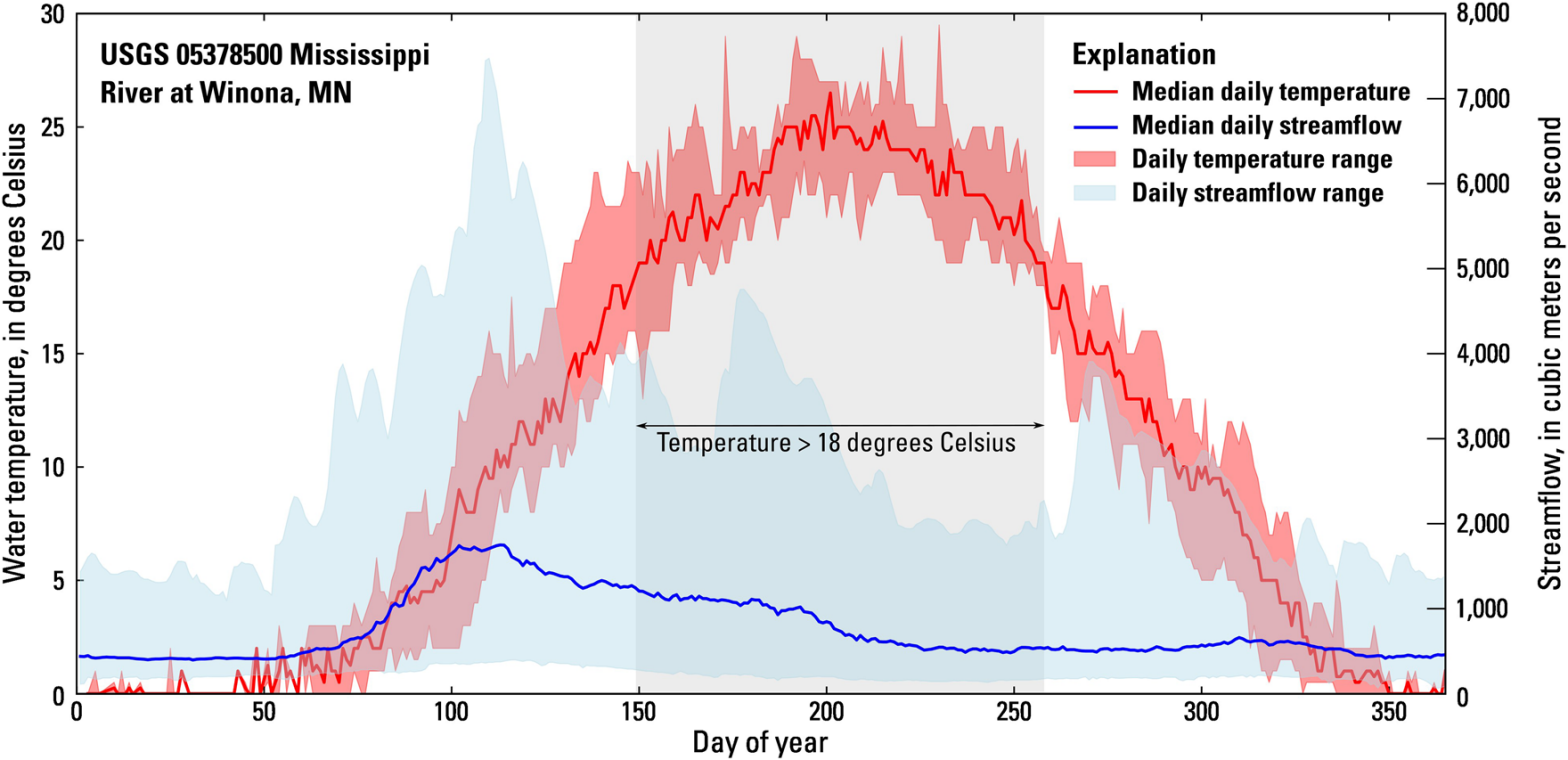
Median and range of daily streamflow and water temperature at U.S. Geological Survey (USGS) streamgage Mississippi River at Winona, MN (05378500). The grey shading shows when the median daily water temperature exceeds 18 degrees Celsius.

The flows used in this study are specified as nominal steady-state streamflows at U.S. Geological Survey (USGS) streamgage 05378500 Mississippi River at Winona, MN (herein referred to as the Winona streamgage), located near the midpoint of the study area in the tailwater of LD 5a (Table 1). The flows were selected based on previous work indicating that invasive carp spawning is associated with high or rising water levels^1^. The lowest nominal flow used in this study (850 cubic meters per second [m^3^/s] or 30,000 cubic feet per second [cfs]) was selected to approximate the annual mean flow at the Winona gage (Table 2)^58^. The highest nominal flow used in this study (5,100 m^3^/s or 180,000 cfs) is equivalent to the 5 percent (%) annual exceedance probability flood at the Winona gage (Table 2)^59^. Although streamflows at the Winona gage can exceed 5,100 m^3^/s, this has historically occurred in mid-spring when water temperatures are still too cold for invasive carp spawning (Fig. 2). During the period of time when water temperatures typically exceed 18 °C (late May to early September), the daily mean streamflow at the Winona gage has ranged from 132.8 to 4,757 m^3^/s (period of record from July 1, 1928, to December 3, 2023)^60^. Therefore, nominal flows greater than 5,100 m^3^/s were considered unlikely to pose a substantial risk for invasive carp spawning and were not included in this study.

**Table 2:** Flow statistics for U.S. Geological Survey streamgage Mississippi River at Winona, MN (05378500). (m^3^/s: cubic meters per second, cfs: cubic feet per second)

Flow Statistic		Flow (m^3^/s)	Flow (cfs)
Annual exceedance probability^a^	1%	6,764	239,000
	2%	6,056	214,000
	4%	5,320	188,000
	5%	5,094	180,000
	10%	4,358	154,000
	20%	3,622	128,000
	50%	2,505	88,500
Mean annual flow^b^		852	30,100

^a^ USACE (2004).

^b^ Granato et al. (2017)

The hypothetical spawning locations were placed in the tailwaters of Lower Saint Anthony Falls (LSAF) LD and LDs 1, 2, 3, 4, 5, 5A, 6, 7, and 8 based on previous work indicating that invasive carp are likely spawning in the tailwaters of lock and dam structures on the Illinois River^21^, Maumee River^23^, and Sandusky River^47^. Except in open-river conditions, dams also present barriers to upstream spawning migrations of invasive carp, thereby inducing aggregation of spawners in tailwaters^57^.

### FluEgg background

FluEgg simulates the biological development and drift of invasive carp eggs and larvae in rivers, from fertilization to the GBI stage, based on user-supplied 1D hydraulic data and water temperature^44,45^. The biological development model within FluEgg tracks how egg density and diameter change over time as a function of water temperature. The user can also specify a constant density and diameter to model any particle with known physical properties. The user-supplied hydraulic data are used to construct a 3D model domain with an idealized 3D velocity field by imposing vertical and transverse velocity profiles based on open channel flow theory and empirically derived parameters. The drift model within FluEgg tracks the movement of each individual egg/larva in the streamwise and lateral directions as a combination of advection (modeled as moving at the same velocity as the water) and diffusion (modeled as a random walk). The motion of the eggs in the vertical direction similarly includes advective and diffusive components (based on the selected eddy diffusivity model) and accounts for the slight negative buoyancy of the eggs. FluEgg predicts the position, extent, and suspended proportion of a plume of fertilized eggs at the time of hatching for a given combination of river flow, water temperature, spawning location, and invasive carp species. After hatching, FluEgg continues to simulate the drift of larvae until they reach the GBI stage, at which point they no longer drift passively and can swim laterally to find nursery habitat^25^.

The channel bed and banks and the water surface are modeled as reflective boundaries, so eggs/larvae cannot leave the channel in the vertical or lateral directions. The hydraulic conditions at the downstream end of the model domain extend infinitely downstream, which allows eggs and larvae to drift out of the model domain, but these drift distances are unreliable. Detailed background on FluEgg and its underlying assumptions can be found in the publications describing the original model and subsequent updates and applications, and the user manual^21,23,41–43,45,48^.

### Hydraulic inputs to FluEgg

The hydraulic inputs for the FluEgg simulations were derived from a pre-existing unsteady-state 1D HEC-RAS model of the UMR that was developed by the USACE as Phase 4 of their UMR hydraulic model update^49^. “Snapshots in time” from this unsteady-state (varying in time) hydraulic model were used as approximations of steady-state (constant in time) hydraulic conditions in the FluEgg simulations. Using a pre-existing hydraulic model allowed for rapid development of the FluEgg simulations in response to management needs without developing, calibrating, and validating a steady-state hydraulic model. The UMR hydraulic model spans from Coon Rapids Dam (0 rkm in this study’s reference system or USACE rm 866.29) to about 29.8 km downstream from LD 10 (434.1 rkm or USACE rm 615). The Minnesota River (up to Savage, MN) and the St. Croix River (up to Stillwater, MN) are explicitly modeled as their own reaches. Other gaged tributaries, including the Chippewa River and Wisconsin River, were modeled as either 1D storage areas or two-dimensional (2D) flow areas. Ungaged inflows were provided by the National Weather Service North Central River Forecast Center. Backwaters behind levees are modeled as 2D flow areas and other backwaters are modeled as 1D storage areas. The model includes calibrated simulations for high flow events in April–May 2001, June–July 2014, and March–June 2019. Full documentation for the UMR hydraulic model can be found in the USACE Upper Mississippi River Phase IV Flood Risk Management Existing Conditions Hydraulic Model Documentation Report^49^.

The rising limb of the March–June 2019 hydrograph was selected to provide the hydraulic inputs for FluEgg because invasive carp spawning has been shown to coincide with high or rising water levels^1^. Time steps were selected such that the modeled flow was within 3% of each of the 9 nominal target flows at the Winona streamgage (Table 1, Supplementary Table 1). The following parameters were exported from the hydraulic model at those time steps: mean depth, streamflow, mean velocity magnitude, and mean shear velocity. Mean cross-sectional lateral and vertical velocities were assumed to be zero throughout the model domain because they cannot be resolved by a 1D hydraulic model. This is justified as the lateral and vertical velocities should average to zero at the reach scale unless water is gained or lost through the boundaries or free surface and flow can only enter and exit a FluEgg cell in the streamwise direction.

The shear velocity exported from the hydraulic model was also used to calculate the Rouse number – a dimensionless parameter that describes the behavior of particles in flowing water – throughout the study area for fertilized, water-hardened eggs under the different simulated flows and temperatures. The Rouse number is defined as $\:P=\:\frac{{w}_{s}}{\kappa\:{u}_{*}}$, where *P* is the Rouse number, $\:{w}_{s}$ is the terminal fall velocity of a particle, $\:\kappa\:$ is the von Kármán constant (0.41), and $\:{u}_{*}$ is the shear velocity of the flow^61^. The terminal fall velocity of fertilized, water-hardened silver carp eggs was determined as a function of temperature^28^(Supplementary Table 2). A Rouse number less than 0.8 indicates that the eggs are completely suspended in the water column with minimal interactions with the bed. As the Rouse number increases from 0.8 to 2.5, the proportion of eggs in suspension decreases, and more interactions with the bed occur. A Rouse number greater than 2.5 indicates that 100% of the eggs are transported as bed load (rolling, sliding, or saltating) or are potentially at rest on the bed.

### FluEgg simulations

FluEgg (version 4.1.1)^44^ was used to simulate the drift of silver carp eggs in the UMR for all 450 scenarios using the hydraulic data extracted from the HEC-RAS model. This study used silver carp eggs/larvae as a proxy for all four invasive carp species, though there are some minor differences in the rate of development and final egg size and density^25–27^. Each simulation included 5,000 silver carp eggs, which were assumed to have been spawned at the water surface and at the midpoint of the channel for each hypothetical spawning location (Supplementary Table 3). The simulations concluded when the larvae reach the GBI stage, at which point they develop the ability to swim laterally and cannot be modelled as passively drifting particles. Eggs/larvae that drifted out of the model domain (drift distances greater than 434.1 km, or 29.8 km downstream from LD 10) were excluded from further analysis. Most of the simulations used a 20-second time step, though a small number used a 19-second time step to satisfy the FluEgg stability criteria for the parabolic-constant eddy diffusivity profile^41^.

The results were exported in FluEgg’s condensed file format, in which the individual egg/larva positions at each time step were summarized by computing the positions of 101 quantiles. The results of these simulations were analyzed to determine the position and extent of the plume of eggs/larvae at hatching time and the time the larvae reach the GBI stage. The plume position and extent in the streamwise direction is summarized in plots using the median position and the span from the 1st percentile to the 99th percentile.

An additional set of FluEgg simulations were run and exported in FluEgg’s “full” file format, which records the position of each egg/larva at each time step, for a selection of 72 scenarios with spawning in the tailwaters of LD 2 and 5. The full file format allows for more detailed analyses of the movements of individual eggs/larvae than the condensed file format, but the file size can be an order of magnitude larger. These additional simulations were run from fertilization until hatching time using a 5-second time step to compare egg transport in Lake Pepin versus through Pools 5A to 10. LD 2 and 5 were chosen as the spawning locations to represent these two reaches, because they enter open-river conditions less frequently than most other dams in the study reach, serving as potential aggregation sites for invasive carp migrating upstream^62^.

The “full” file results were analyzed to determine whether eggs remain in suspension without settling to the bed before hatching. However, defining “settling” is not straightforward with FluEgg results. Ideally, an egg would be considered “settled” if it remained stationary on the bed for some threshold duration. However, in FluEgg, eggs move with the flow and cannot stop and rest on the bed unless the mean water velocity is zero. Even if they approach the bed, they may reflect off or move upwards through the modeled diffusive processes. In this study, “settling” was defined as being within 0.1 m (m) of the bed for at least 80% of a 5-minute time window. This criterion is somewhat arbitrary but was selected such that the results of the settling analysis were consistent with theoretical predictions based on the Rouse number. The suspended hatching rate for each scenario was calculated as the percentage of eggs that did not settle before hatching. Additionally, the position and extent of the plume was recalculated by both halting the settled eggs at the location and time of settling and removing settled eggs from the plume.

## Results

### Hydraulics in Pools 1–10 of the UMR

**Fig. 3:**
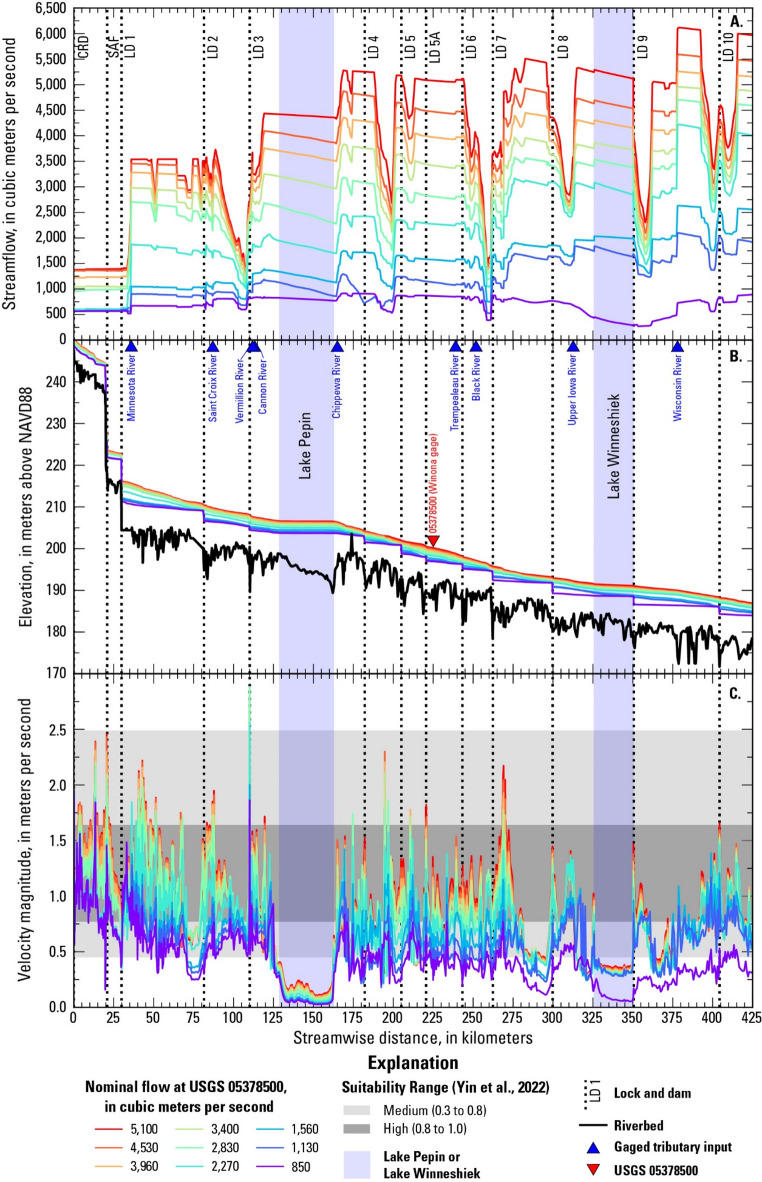
Simulated longitudinal profiles of (**A**) streamflow, (**B**) water surface and riverbed elevations, and (**C**) velocity magnitudes for nine flow scenarios (NAVD88: North American Vertical Datum of 1988).

Longitudinal profiles of water surface elevation, mean velocity, and the local streamflow for the selected nominal flows used in this study demonstrate the hydraulic variability of the study reach (Fig. 3). These results are specific to the time steps pulled from the unsteady-state March–June 2019 simulation^49^. Generally, as the nominal flow increases, the local streamflow also increases, with minor exceptions, such as higher local streamflow near LD 4 for the 850 m^3^/s nominal flow condition compared to 1,130 m^3^/s (Fig. 3A). The reach upstream from the Minnesota River confluence does not show substantial differences in streamflow among the four lowest nominal flow conditions (850 to 2,270 m^3^/s). For a given nominal flow, increases in streamflow with distance downstream along the river result from tributaries and other lateral inflows. Locations with a sharp decrease in local streamflow followed by a sharp increase indicate where water passes from the mainstem river channel into and then back out of backwater or floodplain storage. More gradual decreases in streamflow, in Lake Pepin and Lake Winneshiek for example, may reflect that storage along that stretch of the river was increasing at the selected time step.

Upstream from the Minnesota River confluence, the channel bed and the water surface have a steep “stairstep” profile defined by the hydraulic head across the structures at St. Anthony Falls and LD 1 (Fig. 3B). A gentler “stairstep” shape is also apparent along the rest of the study area in the water surface profiles for the three lowest flows used in this study. At higher flows, the water surface profile becomes smoother as dam gates are raised for open-river conditions^52^. Mean velocities vary throughout the study reach (Fig. 3C) due to the influence of the LDs, tributary inputs, and variations in conveyance. Generally, velocities increase with increasing flow; exceptions occur where rising water levels activate side channels, flow into backwater storage areas, and/or flow onto the floodplain. Mean velocities in Lake Pepin are consistently slow compared to the rest of the study area, averaging 0.06 m/s at 850 m^3^/s and 0.19 m/s at 5,100 m^3^/s. The downstream reaches of Pools 2, 8, and 9 (Lake Winneshiek) are also relatively slow-flowing compared to the surrounding areas.

**Fig. 4:**
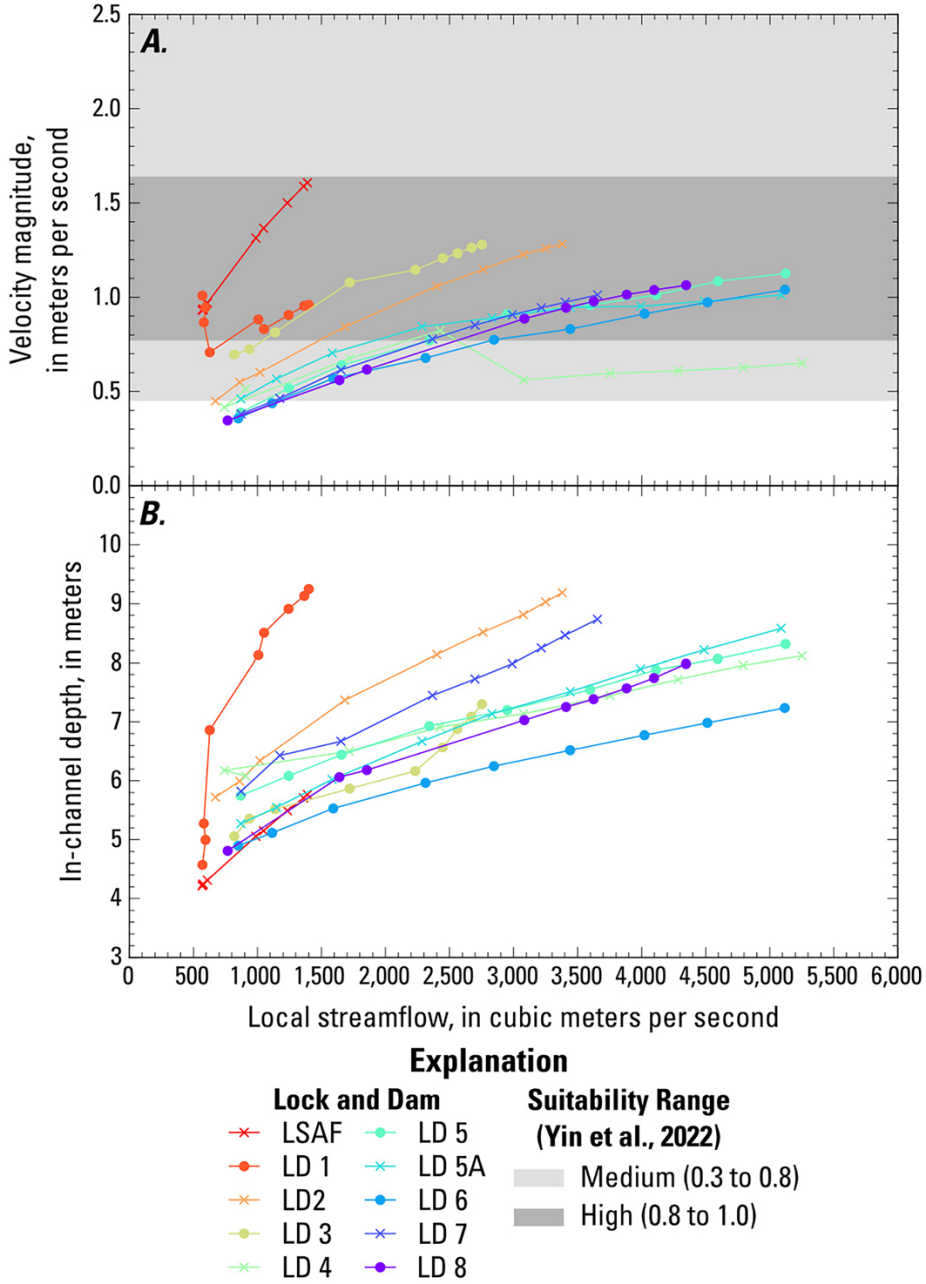
(**A**) Velocity magnitude and (**B**) in-channel depth versus the local streamflow in lock and dam tailwaters in the study area, overlain with the range in velocities with suitability value of 0.3 to 0.8 (medium suitability) and greater than 0.8 (high suitability) from Yin et al. (2022). (LSAF: Lower Saint Anthony Falls, LD: Lock and Dam)

The velocity and depth were averaged over a 2-km reach downstream from each LD to examine the hydraulics in the hypothetical spawning locations (Fig. 4). Local streamflows were used for this analysis as they better represent local site conditions than the nominal flow conditions. Velocities increase with increasing flow in the tailwaters of LSAF LD and LDs 2, 3, 5, 5A, 6, 7, and 8. The relation between mean velocity and flow is more complex for the tailwaters of LD 1 and LD 4. In the tailwater of LD 1, simulated operations of the gates at LD 2 affect the relation between mean velocity and flow. As the nominal flow increases from 850 to 2,270 m^3^/s, the local streamflow in the LD 1 tailwater increases from 570 to 630 m^3^/s. During this time, the LD 2 gates are partially open, and Pool 2 begins to fill, resulting in an increase in depth from 4.6 m to 6.9 m and a decrease in mean velocity from 1.0 to 0.7 m/s. As the nominal flow increases from 2,270 to 2,830 m^3^/s, the remaining LD 2 gates fully open. In the tailwater of LD 4, as the nominal flow increases from 2,270 to 2,830 m^3^/s (local flow 2,430 to 3,080 m^3^/s), the side channels transition from stagnant to flowing. This results in an increase in the cross-sectional area of the flow and a decrease in the mean velocity.

The spawning suitability index (SSI) developed by Yin et al. (2022)^24^ provides some context for the mean velocities in the study area. “Medium” suitability for initiation of spawning is defined as SSI between 0.3 and 0.8, and “high” suitability is defined as SSI greater than 0.8, following Jackson et al. (2024)^23^. Upstream from Lake Pepin, mean velocities fall within the medium to high range of spawning suitability for all the flows, with a few minor exceptions, including the low velocity zone in Pool 2 at less than 1,560 m^3^/s (Fig. 3C). Downstream from Lake Pepin, the mean velocities are also in the medium to high SSI range, except at 850 m^3^/s and in the low velocity zones in Pools 8 and 9 (Lake Winneshiek). Velocities within Lake Pepin fall well below the range for medium spawning suitability. The velocities in the hypothetical spawning locations are generally in the medium to high SSI range, except for some of the lower flows at LDs 4–8 (Fig. 4A).

**Fig. 5:**
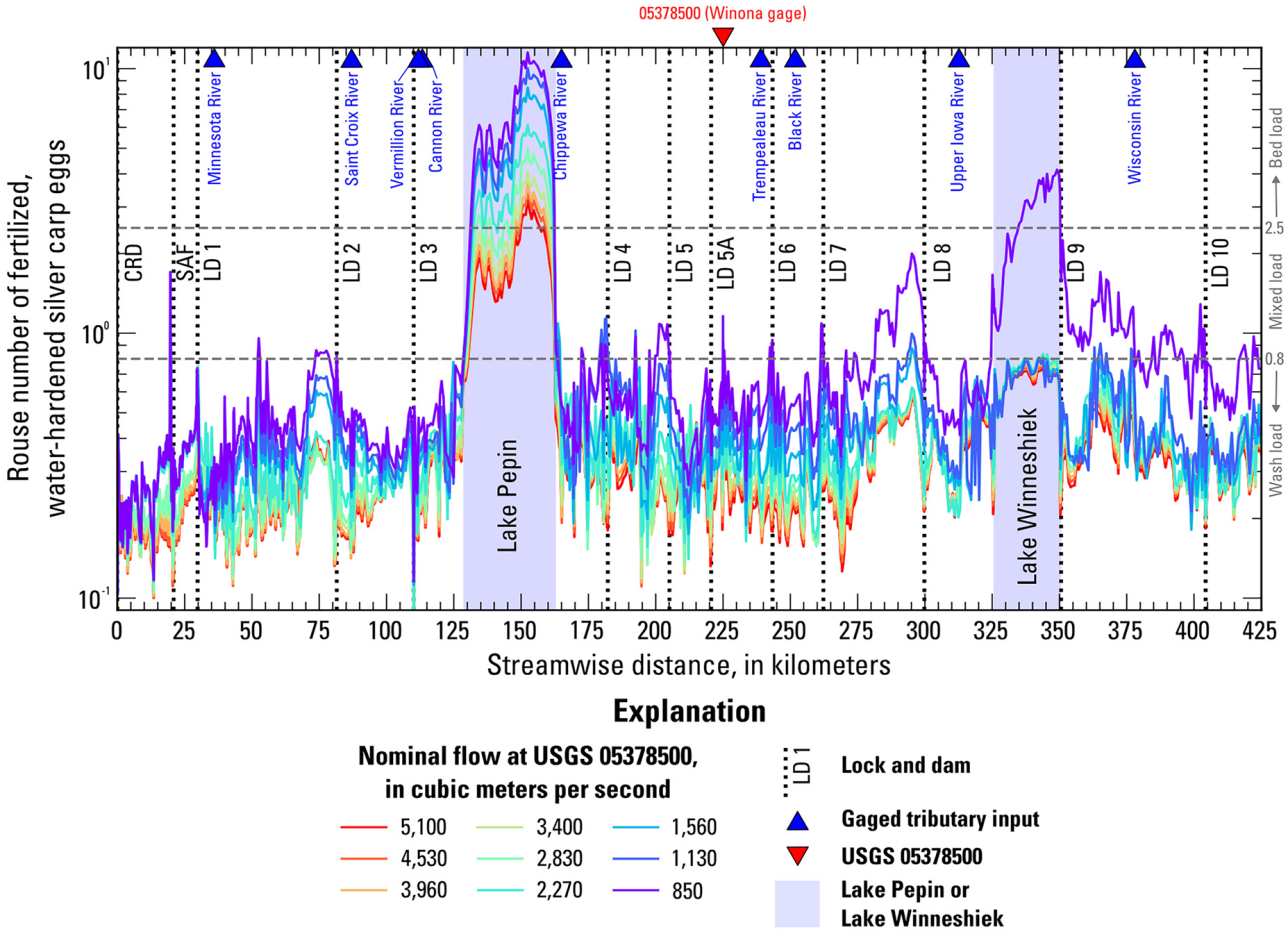
Simulated longitudinal profile of the Rouse number for fertilized, water-hardened silver carp eggs at 22 degrees Celsius for nine flow scenarios. The thresholds for transport as wash load (< 0.8), mixed load (0.8–2.5), and bed load (> 2.5) are shown as dashed gray lines.

Profiles of the Rouse number show the theoretical capacity of the flow to transport fertilized water-hardened silver carp eggs in suspension (Fig. 5). A Rouse number below 0.8 indicates that the eggs would be fully in suspension, the transitional range of 0.8 to 2.5 indicates mixed transport, and values above 2.5 indicate bed load transport or no movement. According to the Rouse number, silver carp eggs would be transported in suspension throughout nearly the entire study area except for Lake Pepin for most of the flows examined in this study. The Rouse number indicates mixed transport for the 850 m^3^/s flow (and to a lesser extent, 1,130 m^3^/s) in scattered locations besides Lake Pepin. Within Lake Winneshiek, the Rouse number indicates both mixed transport and bed load transport of silver carp eggs for the 850 m^3^/s flow.

Lake Pepin can be divided into two sections based on the Rouse number. In the upstream section of Lake Pepin, the Rouse number indicates mixed load transport for nominal flows that are greater than 2,830 m^3^/s and bed load transport for flows less than 2,830 m^3^/s. For the 2,830 m^3^/s nominal flow, the Rouse number fluctuates above and below the 2.5 threshold. Conversely, in at least part of the downstream section of Lake Pepin the Rouse number exceeds 2.5 for all the flows, indicating bed load transport of silver carp eggs. The survival rate of silver carp eggs transported as bed load is unknown, but transport as bed load is expected to cause damage from impacts with the bed, abrasion with sediment, and high turbulence.

### Egg and larval drift results

**Fig. 6:**
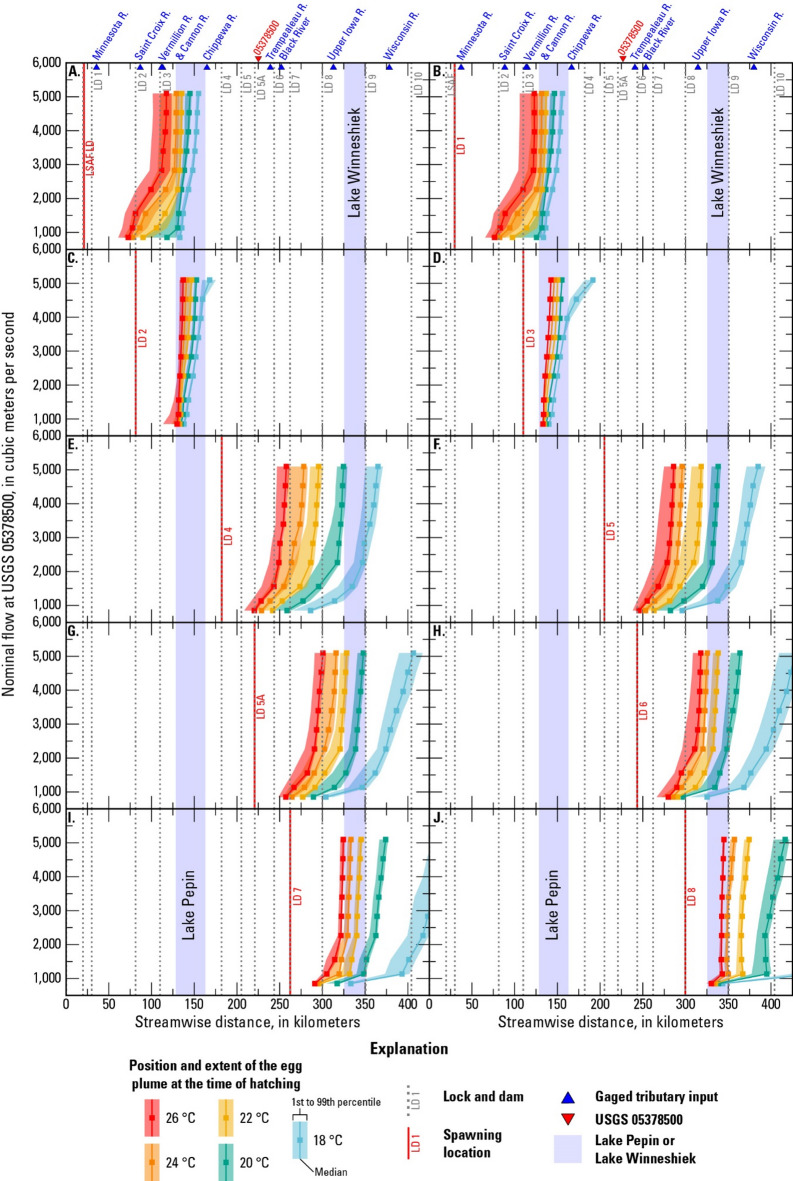
Location of the egg plume at hatching time for different nominal flows and temperatures and for spawning in the tailwater of (**A**) Lower Saint Anthony Falls (LSAF) Lock and Dam (LD), (**B**) LD 1, (**C**) LD 2, (**D**) LD 3, (**E**) LD 4, (**F**) LD 5, (**G**) LD 5A, (**H**) LD 6, (**I**) LD 7, and (**J**) LD 8. (R.: River, °C: degrees Celsius)

**Fig. 7:**
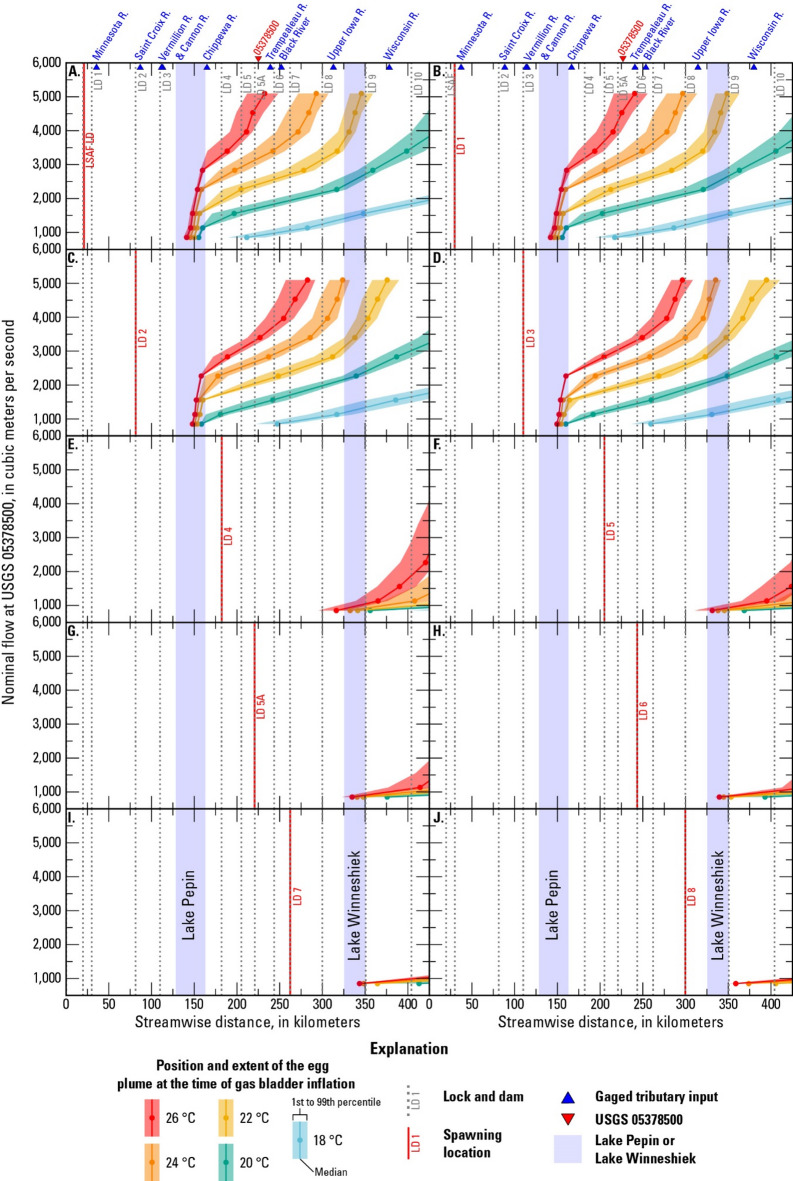
Location of the larval plume at the time of gas bladder inflation for different nominal flows and temperatures and for spawning in the tailwater of (**A**) Lower Saint Anthony Falls (LSAF) Lock and Dam (LD), (**B**) LD 1, (**C**) LD 2, (**D**) LD 3, (**E**) LD 4, (**F**) LD 5, (**G**) LD 5A, (**H**) LD 6, (**I**) LD 7, and (**J**) LD 8. (R.: River, °C: degrees Celsius)

The location and extent of the egg/larval plume at key developmental stages – hatching and GBI – depends on the hypothetical spawning location, water temperature (controls drift time), and flow (controls drift speed) (Figs. 6 and 7). The drift results described in this section do not account for potential mortality of eggs/larvae. For spawning in the tailwater of LSAF and LD 1, warmer temperatures and lower flows promote hatching upstream from Lake Pepin, whereas cooler temperatures and higher flows result in the eggs hatching within Lake Pepin (Fig. 6). In contrast, nearly all the scenarios with spawning in the tailwaters of LDs 2 and 3 result in eggs hatching within Lake Pepin (Fig. 6). The eggs that hatch in or drift through Lake Pepin likely undergo extended transport as bedload (Fig. 5). Assuming the eggs survive to hatch into larvae, the larvae spawned at LSAF and LDs 1–3 reach the GBI stage within the study area for most scenarios, except under relatively low temperatures and high flows (Fig. 7).

For spawning in the tailwaters of LDs 4, 5, and 5A, the eggs hatch within the study area (Fig. 6). Eggs spawned in the tailwaters of LDs 6, 7, and 8 hatch within the study area for water temperatures in the 20 to 26 °C range, but can drift out of the study area if flow is high enough and the water temperature is 18 °C (Fig. 6). After hatching, the larvae spawned at LDs 4–8 drift out of the study area before reaching the GBI stage, except for relatively warm temperatures and low flows (Fig. 7).

### Suspension of eggs prior to hatching

**Fig. 8:**
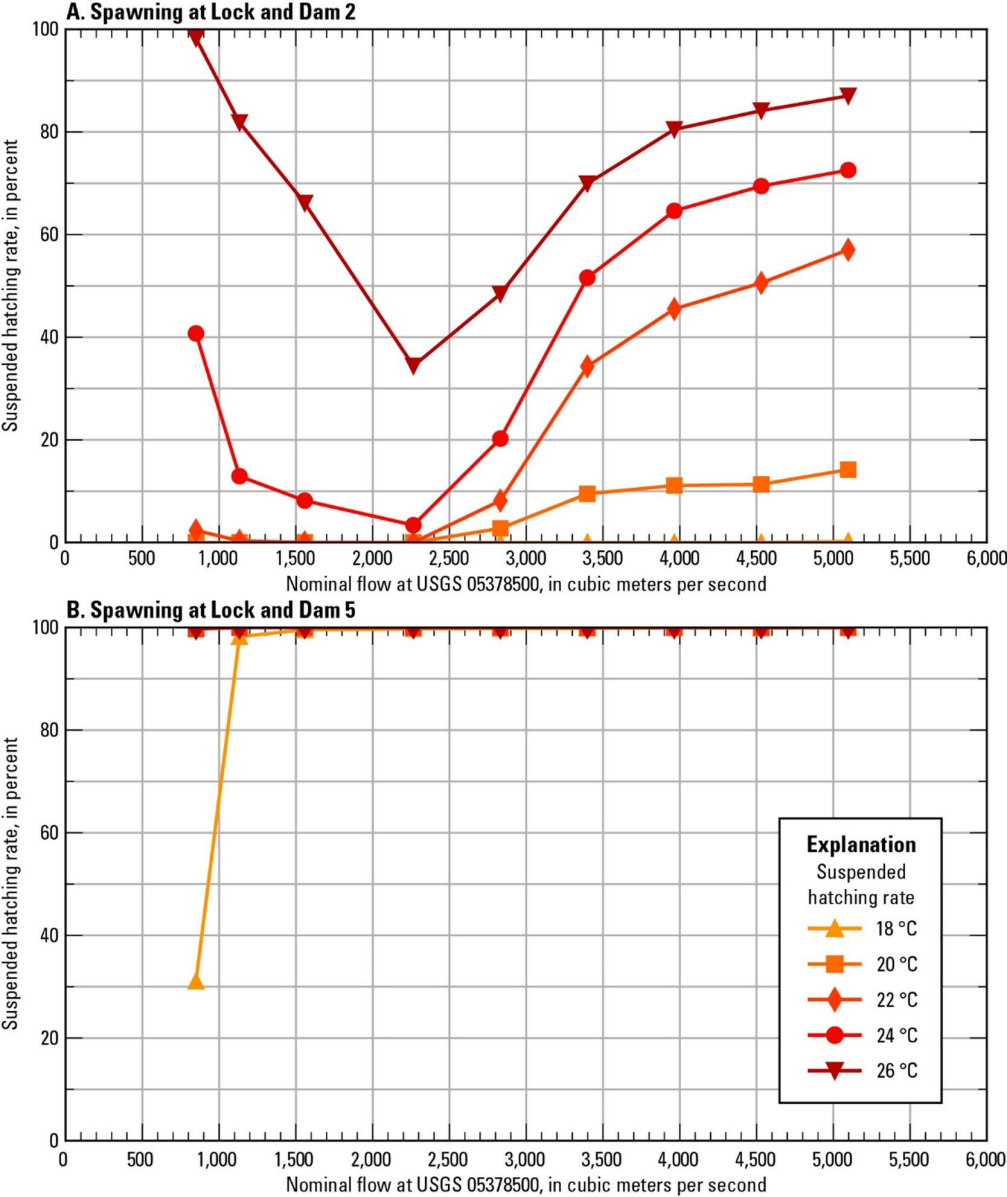
Suspended hatching rate for different nominal flows and water temperatures and for spawning in the tailwater of (**A**) Lock and Dam 2 and (**B**) Lock and Dam 5. (°C: degrees Celsius)

The suspended hatching rate (percent of eggs that did not settle prior to hatching) was calculated for selected scenarios with spawning in the tailwaters of LD 2 and LD 5 (Fig. 8, Supplementary Table 3). The eggs spawned in the tailwater of LD 2 drift through Lake Pepin, whereas the eggs spawned in the tailwater of LD 5 drift through Pools 5A – 10 (Fig. 6C, F). For spawning in the tailwater of LD 5, the suspended hatching rate is at least 98.2% for all scenarios except 850 m^3^/s at 18 °C, which is consistent with the Rouse number profiles (Fig. 8). At 18 °C and 850 m^3^/s, eggs spawned below LD 5 pass through most of Pool 8, and hatch where the Rouse number indicates mixed transport for that flow (Figs. 5 and 6F).

For spawning in the tailwater of LD 2, the suspended hatching rate is 0.0% to 0.2% for all the flows at 18 °C (Fig. 8). The time it takes for the eggs to hatch at 18 °C is long enough that, for all flows, the plume passes through a part of Lake Pepin in which the Rouse number predicts bedload transport or settling (Figs. 5 and 6C). The suspended hatching rate at 22 °C is less than 3% for flows up to 2,270 m^3^/s and increases from 8.2% at 2,830 to 57.0% at 5,100 m^3^/s. The eggs develop faster at 22 °C than 18 °C, and the plume only moves through the upstream section of Lake Pepin, where bed load transport or no movement is expected for flows up to 2,270 m^3^/s and mixed transport is expected for flows 2,830 m^3^/s and above. Finally, at 26 °C the suspended hatching rate decreases from 98.2% to 34.4% from 850 to 2,270 m^3^/s, as the increasing velocity of the flow results in more and more of the plume entering Lake Pepin before hatching. The suspended hatching rate at 26 °C then increases from 69.9% to 87.0% as flow increases from 2,830 to 5,100 m^3^/s, resulting in increased turbulence and mixed transport.

**Fig. 9:**
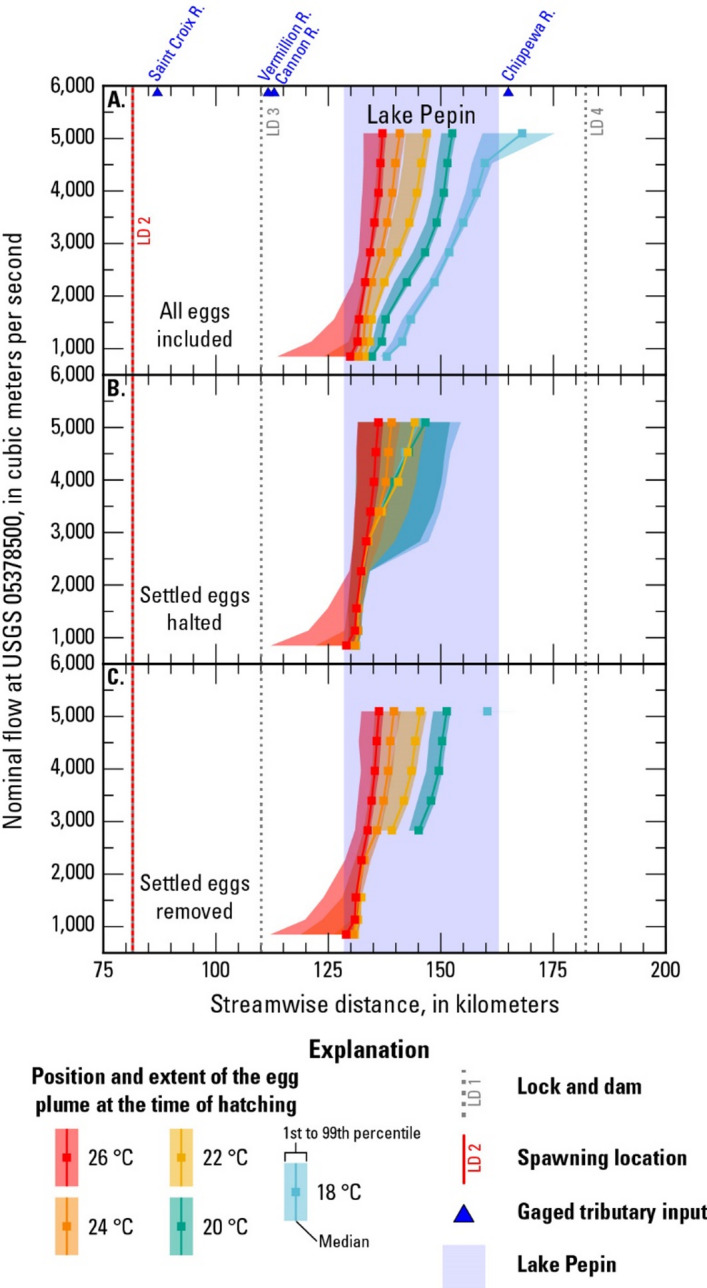
Location of the egg plume at the time of hatching for different flows and temperatures and for spawning in the tailwater of Lock and Dam 2 with (**A**) all eggs included, (**B**) settled eggs halted at the position of settling, and (**C**) settled eggs removed from the plume (R.: River, °C: degrees Celsius).

The position and extent of the egg plumes at hatching time were recalculated for the scenarios with spawning in the tailwater of LD 2 by (1) halting settled eggs at the time and location of settling and (2) by removing settled eggs from the plume. Halting the settled eggs results in an upstream shift in the hatching position for nearly all the modeled scenarios with spawning at LD 2, and the effect is more pronounced for scenarios with lower suspended hatching rates (Fig. 9A, B). The results are similar for removing settled eggs from the plume, with the major differences being that the scenarios with zero suspended hatching do not show up on the plot at all and the extent of the plume is narrower compared to the “halted” case (Fig. 9C). For the scenario with 5,100 m^3^/s and 18 °C, the few remaining eggs (*n* = 48) in the “removed” case are further downstream than the plotted extent of the plume in the “halted” case, as they are not within the 1st to 99th percentile of the “halted” case plume.

## Discussion

Results from 450 FluEgg simulations indicate that the relative recruitment risk in Pools 1–10 of the UMR is largely controlled by whether spawning occurs upstream or downstream from Lake Pepin, though streamflow and water temperature also play a role. Here, we assume that eggs that settle to the bed are less likely to survive than eggs that remain suspended until hatching and that larvae will contribute to recruitment in the pool where they reach the GBI stage. Invasive carp larvae leave the drift to seek out nursery habitat upon reaching the GBI stage, when they develop the ability to swim laterally and must begin to exogenously feed^33^. We did not examine the movement of post-GBI larvae because the drift processes simulated in FluEgg are purely physical. Simulating the movement of post-GBI larvae would require a model that incorporates larval behavior and swimming ability. It is also possible for larvae that drift out of the study area to return as mature fish, but recruitment by immigration is beyond the scope of this study. Our hypothetical scenarios assume spawning is occurring in the tailwaters of the locks and dams on the mainstem of the UMR, though it is possible that tributaries such as the Chippewa, Black, Wisconsin, Root, St. Croix, and Minnesota Rivers could provide spawning habitat for invasive carp^63,64^. Additional FluEgg simulations with spawning in the tributaries could show how this additional drift distance affects the likelihood of recruitment in the study area^65^.

For scenarios with spawning downstream from Lake Pepin, the larvae do not reach the GBI stage until they are downstream from LD 10 (and beyond the model domain) for most of the simulated scenarios. Recruitment would therefore occur downstream from the study area for scenarios with spawning downstream from Lake Pepin. The scenarios that resulted in at least part of the plume reaching GBI in the study area were generally those with lower streamflows, higher temperatures (shorter drift times), and spawning further upstream.

For scenarios with spawning upstream from Lake Pepin, the larvae reach the GBI stage within the model domain for most scenarios, assuming survival and development to this stage. However, the in-depth analysis of egg settling potential in Lake Pepin for spawning at LD 2 and the longitudinal profiles of the Rouse number indicate that settling is likely for eggs that drift through Lake Pepin. Spawning closer to Lake Pepin and at cool temperatures (longer drift times before hatching) means it is more likely the eggs will enter the lake and move into an area where they will settle. Higher flows result in greater turbulence, promoting suspension, but also increase the likelihood that the eggs will reach Lake Pepin where they will either be transported as bedload or settle.

Several factors contribute to egg and larval mortality besides settling. In general, the high fecundity of broadcast spawners is balanced by high mortality^66^. Damage from turbulence or abrasion by suspended sediment^30^ as well as predation may contribute to mortality. Additionally, competition can lead to mortality for post-GBI larvae either through starvation or negative effects on development^67,68^. The numerous side channels and backwaters in the upper impounded reach of the UMR would likely provide abundant nursery habitat for exogenously feeding invasive carp larvae and young fish, as they have for the larvae and juveniles of other species^69^. However, the extent to which competition for resources in nursery habitat could affect the survival of exogenously feeding invasive carp larvae in the UMR is unknown. In general, mortality is not modeled in FluEgg^48^ due to insufficient quantitative data on the mortality rates of invasive carp eggs and larvae.

Although spawning has not yet been confirmed in Pools 1–10 of the UMR, observations of invasive carp movements during a recent high flow event provide some context for the results of this study. Moderate to major flooding occurred on the UMR in the spring of 2023, with flood peaks setting top 5 records at gages from Wabash, MN, to Guttenberg, Iowa (IA)^70^. Dams along the UMR were fully opened to pass the floodwaters, resulting in a period of simultaneous open-river conditions at LDs 18 through 5A in April 2023^57^. During this period, acoustic telemetry data showed bighead and silver carp from below LD 15 moved upstream, including 42 tagged fish that passed upstream into Pool 8 and 10 tagged fish that passed upstream into Pool 5A, where they stopped, most likely due to the closure of LD 5^57^. By late May 2023, water temperatures measured at LD 5 warmed to above 18 °C^71^ and flows were still high (approx. 2,270 m^3^/s on May 22, 2023, peak of 2,600 m^3^/s on May 24, 2023, at the Winona gage). Conditions were likely suitable for the invasive carp in Pool 5A to spawn in the tailwater of LD 5. Had spawning occurred in the tailwater of LD 5 at this time, the eggs would be expected to hatch in Pool 10, though the larvae would drift out of the study area (below LD 10) before reaching the GBI stage. No invasive carp eggs or larvae were detected in light trap samples taken in Pool 5A in 2023^39^, though light traps were not the ideal gear for detecting spawning at this location given the predicted hatching location in Pool 10. Future sampling can benefit from this study to site ichthyoplankton tows (an active gear that can capture drifting eggs) at potential successful spawning sites and light traps (a passive gear that requires larval fish to swim into the trap) at potential hatching and GBI sites.

The upstream migration of tagged bighead and silver carp in the UMR in spring 2023 highlights the possibility that large numbers of invasive carp may move upstream over long distances (mean: 270 rkm; range: 48–381 rkm; *n* = 53^57^ during open-river conditions, posing a risk for reproduction in the study area. Fish that moved upstream during the spring 2023 flood may be able to move even further upstream during the next open-river event, posing a potential risk for recruitment in the study area, should spawning occur above Lake Pepin. For example, a tagged silver carp that overwintered in Pool 5A in 2022–2023 passed upstream through LD 5 while it was still open and made its way to Pool 3 and the St. Croix River^6^. Many invasive carp were still present in Pool 6 by November and December 2023, when the Minnesota and Wisconsin Departments of Natural Resources removed 408 invasive carp from that pool^6^. However, early telemetry data suggest that many invasive carp move downstream out of the study area during the winter months^39^. Seasonality in upstream movements, closely linked to flooding and open-river conditions^57^, is therefore an important driver of recruitment risk in this area.

The limitations of FluEgg have been described in the many previous applications of the model^21,23,40–42,46–48,65^. FluEgg uses a rectangular model channel and an idealized 3D velocity field built from a 1D hydraulic model to represent the highly complex systems of the UMR. The UMR contains side channels, meanders, braid bars, islands, backwaters, and navigation structures, all of which increase the three-dimensionality of the flow structure. Previous 3D modeling studies have shown that the 3D flow structure arising from complex geomorphology and river-training structures affects the drift pathways and likelihood of entrapment or settling of invasive carp eggs^17,72,73^. As a result, FluEgg simulations likely underestimate dispersion and residence time of eggs/larvae due to eggs/larvae that get trapped in backwaters or other low-velocity zones, move slowly through side channels, or otherwise have their drift affected by the 3D velocity field^17,72,73^. Similarly, eggs and larvae may become trapped or delayed in recirculating flows or stagnant zones around lock and dam structures. The suspension dynamics and mortality rates of eggs would also be affected by the increased turbulence and the highly 3D flow structure around locks and dams, particularly around the dam gates^74^. The locks and dams in Pools 20–26 of the UMR may be hindering recruitment in those pools, though this has yet to be shown definitively^75^.

Another limitation of this study was the use of “snapshot in time” hydraulic conditions and constant water temperatures. Streamflow and water temperature both vary in time and space during real spawning events, in response to the passage of flood waves and varying lateral inputs. Water temperature also varies in response to changing air temperature, which includes a diurnal component. Changing hydraulic conditions affect the 3D flow structure^73^ and lock and dam operations, which affect the rate of drift of the eggs/larvae and the likelihood of settling of the eggs. Changes in water temperature affect the rate of biological development of the eggs/larvae, as warmer temperatures result in more rapid development^25–28^. Simulating changing flow and temperature conditions would be more realistic but requires selection of a particular flow event and the timing of the spawning event. This greatly increases the number of possible scenarios and the complexity of the study design. In the context of invasive carp in the UMR, the urgency of the management needs justified the “snapshot” approach used herein, despite the potential added uncertainty.

Despite its limitations, FluEgg has been used to successfully predict the potential for spawning and the locations of spawning areas in both the Maumee and Sandusky Rivers^23,40,46,76^ and successfully replicated most characteristics of egg-age variability in field ichthyoplankton samples^47^. Further validation of FluEgg could be provided through applications focused on native pelagic spawners, such as freshwater drum. Although previous 3D drift modeling studies have greatly advanced our understanding of how and where eggs/larvae may become trapped or settle, these studies were limited to much smaller study reaches compared to the present study^17,72,73^. It is not currently feasible to simulate the hydrodynamics and egg/larval drift dynamics in 3D at the scale of multiple pools in the UMR. A reduced-complexity model like FluEgg can handle an expansive study reach and allows for assessment of many potential spawning scenarios, providing critical information to managers making decisions about where, when, and how to sample for early life stages of invasive carp. The management of invasive carp, and invasive species more generally, likely benefits from combining insights from reduced complexity models with those from highly detailed models, that may be more limited in scope and scale.

## Conclusions

Managing the growing population of invasive carp in the UMR relies on monitoring for all life stages to understand how the abundance, demographics, and distributions of these species are changing over time^37^. Monitoring and response actions are sited based on the best available information on invasive carp behavior, captures, telemetry, sightings, and environmental DNA detections. However, it is currently unknown where invasive carp could successfully recruit in the UMR if a spawning event were to occur. In this study, simulations of invasive carp egg and larval drift were used to assess the risk of recruitment in Pools 1–10 of the UMR for various hypothetical spawning scenarios. The results indicate that eggs spawned upstream from Lake Pepin are likely to settle in the lake before hatching under a wide variety of river conditions (flow and water temperature). Additionally, for many of the scenarios with spawning downstream from Lake Pepin, larvae drift downstream and out of the study area (Pools 1–10) before they reach gas bladder inflation and leave the drift to seek out nursery habitat and food. However, there are some scenarios that may result in recruitment within the study area for spawning further upstream, at lower flows, and at higher temperatures (shorter drift times). The results of this study can be used to inform gear and site selection to improve the likelihood of detecting any spawning occurring within the study reach and to target management actions to reduce invasive carp abundance in areas most likely to support recruitment. Although the FluEgg simulations simplify a highly 3D system, the simplicity of the model allowed for testing a wide range of hypothetical spawning scenarios over a long study reach. Thus, this study exemplifies the benefits of reduced complexity modeling as a tool for invasive species management.

## Supplementary Information

Below is the link to the electronic supplementary material.

Supplementary Material 1

## Data Availability

The data and simulation results that support the findings of this study can be obtained through USGS ScienceBase at: [https://doi.org/10.5066/P1TGKMRJ].
