# Supplementary material for: Drift and dispersion of silver carp (Hypophthalmichthys molitrix) eggs and larvae for hypothetical spawning scenarios in the Upper Mississippi River
Source: Sci Rep. 2026 May 6;16:14421. doi: 10.1038/s41598-026-41803-w (PMC13149695; doi:10.1038/s41598-026-41803-w)
Supplement: Supplementary file 1 — Supplementary Material 1 [file 41598_2026_41803_MOESM1_ESM.docx]

Supplementary Tables

Supplementary Table 1: Hydraulic model time steps corresponding to the target flows for FluEgg simulations. (m^3^/s: cubic meters per second, %: percent)

| Target flow^a^ (m^3^/s) | Time step^b^ | Simulated flow^a^ (m^3^/s) | Percent difference^c^ | Absolute difference (m^3^/s) |
| --- | --- | --- | --- | --- |
| 850 | 13MAR2019​ 0000 | 868 | 2.1% | 18 |
| 1,130 | 16MAR2019​ 0600 | 1,131 | 0.1% | 1 |
| 1,560 | 18MAR2019​ 0600 | 1,574 | 0.9% | 14 |
| 2,270 | 23MAR2019​ 1200 | 2,271 | 0.1% | 1 |
| 2,830 | 25MAR2019​ 1200 | 2,808 | -0.8% | -22 |
| 3,400 | 27MAR2019​ 0000 | 3,416 | 0.5% | 16 |
| 3,960 | 28MAR2019​ 1800 | 3,970 | 0.2% | 10 |
| 4,530 | 30MAR2019​ 0600 | 4,467 | -1.4% | -63 |
| 5,100 | 01APR2019​ 0600 | 5,077 | -0.5% | -23 |

^a^ At U.S. Geological Survey streamgage Mississippi River at Winona, MN (05378500)

^b^ Time step format DDMMMYYYY hhmm

^c^ Percent difference = 100 x (Simulated – Target)/Target

Supplementary Table 2: Density and terminal fall velocity of fertilized, water-hardened silver carp eggs at water temperatures ranging from 18 to 28 degrees Celsius (from George et al., 2017). (°C: degrees Celsius, kg/m^3^: kilograms per cubic meter, m/s: meters per second)

| Temperature (°C) | Density (kg/m^3^) | Terminal fall velocity (m/s) |
| --- | --- | --- |
| 18 | 1,000.1258 | 0.6227 |
| 19 | 999.9194 | 0.6251 |
| 20 | 999.7129 | 0.6307 |
| 21 | 999.5065 | 0.6394 |
| 22 | 999.3000 | 0.6514 |
| 23 | 999.0935 | 0.6665 |
| 24 | 998.8871 | 0.6848 |
| 25 | 998.6806 | 0.7062 |
| 26 | 998.4742 | 0.7306 |
| 27 | 998.2677 | 0.7580 |
| 28 | 998.0612 | 0.7883 |

Supplementary Table 3: Results of the settling analysis for selected scenarios with spawning at Lock and Dam 2 and 5, with settling defined as being within 0.1 meter of the bed for at least 80% of a 5-minute time window. Each simulation began with 5,000 fertilized eggs. (m^3^/s: cubic meters per second, °C: degrees Celsius, LD: Lock and Dam, %: percent)

| **Spawning Location** | **Flow (m^3^/s)** | **Water Temperature (°C)** | **Hatching time (hours)** | **Number of settled eggs** | **Settling rate** | **Suspended hatching rate** |
| --- | --- | --- | --- | --- | --- | --- |
| LD 2 | 850 | 18 | 63.55 | 5,000 | 100.0% | 0.0% |
| LD 2 | 1,130 | 18 | 63.55 | 5,000 | 100.0% | 0.0% |
| LD 2 | 1,560 | 18 | 63.55 | 5,000 | 100.0% | 0.0% |
| LD 2 | 2,270 | 18 | 63.55 | 5,000 | 100.0% | 0.0% |
| LD 2 | 2,830 | 18 | 63.55 | 5,000 | 100.0% | 0.0% |
| LD 2 | 3,400 | 18 | 63.55 | 5,000 | 100.0% | 0.0% |
| LD 2 | 3,960 | 18 | 63.55 | 5,000 | 100.0% | 0.0% |
| LD 2 | 4,530 | 18 | 63.55 | 4,999 | 100.0% | 0.0% |
| LD 2 | 5,100 | 18 | 63.55 | 4,952 | 99.8% | 0.2% |
| LD2 | 850 | 20 | 44.25 | 5,000 | 100.0% | 0.0% |
| LD2 | 1,130 | 20 | 44.25 | 5,000 | 100.0% | 0.0% |
| LD2 | 1,560 | 20 | 44.25 | 5,000 | 100.0% | 0.0% |
| LD2 | 2,270 | 20 | 44.25 | 5,000 | 100.0% | 0.0% |
| LD2 | 2,830 | 20 | 44.25 | 4,862 | 97.2% | 2.8% |
| LD2 | 3,400 | 20 | 44.25 | 4,526 | 90.5% | 9.5% |
| LD2 | 3,960 | 20 | 44.25 | 4,445 | 88.9% | 11.1% |
| LD2 | 4,530 | 20 | 44.25 | 4,434 | 88.7% | 11.3% |
| LD2 | 5,100 | 20 | 44.25 | 4,290 | 85.8% | 14.2% |
| LD 2 | 850 | 22 | 32.89 | 4,867 | 97.6% | 2.4% |
| LD 2 | 1,130 | 22 | 32.89 | 4,981 | 99.7% | 0.3% |
| LD 2 | 1,560 | 22 | 32.89 | 4,997 | 100.0% | 0.0% |
| LD 2 | 2,270 | 22 | 32.89 | 4,997 | 100.0% | 0.0% |
| LD 2 | 2,830 | 22 | 32.89 | 4,192 | 91.8% | 8.2% |
| LD 2 | 3,400 | 22 | 32.89 | 2,618 | 65.7% | 34.3% |
| LD 2 | 3,960 | 22 | 32.89 | 2,039 | 54.5% | 45.5% |
| LD 2 | 4,530 | 22 | 32.89 | 1,853 | 49.4% | 50.6% |
| LD 2 | 5,100 | 22 | 32.89 | 1,618 | 43.0% | 57.0% |
| LD2 | 850 | 24 | 25.87 | 2,963 | 59.3% | 40.7% |
| LD2 | 1,130 | 24 | 25.87 | 4,354 | 87.1% | 12.9% |
| LD2 | 1,560 | 24 | 25.87 | 4,592 | 91.8% | 8.2% |
| LD2 | 2,270 | 24 | 25.87 | 4,831 | 96.6% | 3.4% |
| LD2 | 2,830 | 24 | 25.87 | 3,988 | 79.8% | 20.2% |
| LD2 | 3,400 | 24 | 25.87 | 2,420 | 48.4% | 51.6% |
| LD2 | 3,960 | 24 | 25.87 | 1,769 | 35.4% | 64.6% |
| LD2 | 4,530 | 24 | 25.87 | 1,528 | 30.6% | 69.4% |
| LD2 | 5,100 | 24 | 25.87 | 1,372 | 27.4% | 72.6% |
| LD 2 | 850 | 26 | 21.35 | 58 | 1.8% | 98.2% |
| LD 2 | 1,130 | 26 | 21.35 | 706 | 18.2% | 81.8% |
| LD 2 | 1,560 | 26 | 21.35 | 1,406 | 33.9% | 66.1% |
| LD 2 | 2,270 | 26 | 21.35 | 2,879 | 65.6% | 34.4% |
| LD 2 | 2,830 | 26 | 21.35 | 2,084 | 51.6% | 48.4% |
| LD 2 | 3,400 | 26 | 21.35 | 1,082 | 30.1% | 69.9% |
| LD 2 | 3,960 | 26 | 21.35 | 656 | 19.5% | 80.5% |
| LD 2 | 4,530 | 26 | 21.35 | 520 | 15.9% | 84.1% |
| LD 2 | 5,100 | 26 | 21.35 | 416 | 13.0% | 87.0% |
| LD 5 | 850 | 18 | 63.55 | 2,840 | 68.9% | 31.1% |
| LD 5 | 1,130 | 18 | 63.55 | 54 | 1.8% | 98.2% |
| LD 5 | 1,560 | 18 | 63.55 | 8 | 0.4% | 99.6% |
| LD 5 | 2,270 | 18 | 63.55 | 5 | 0.3% | 99.7% |
| LD 5 | 2,830 | 18 | 63.55 | 6 | 0.2% | 99.8% |
| LD 5 | 3,400 | 18 | 63.55 | 3 | 0.2% | 99.8% |
| LD 5 | 3,960 | 18 | 63.55 | 2 | 0.1% | 99.9% |
| LD 5 | 4,530 | 18 | 63.55 | 3 | 0.1% | 99.9% |
| LD 5 | 5,100 | 18 | 63.55 | 0 | 0.1% | 99.9% |
| LD 5 | 850 | 22 | 32.89 | 11 | 0.3% | 99.7% |
| LD 5 | 1,130 | 22 | 32.89 | 1 | 0.0% | 100.0% |
| LD 5 | 1,560 | 22 | 32.89 | 1 | 0.0% | 100.0% |
| LD 5 | 2,270 | 22 | 32.89 | 0 | 0.0% | 100.0% |
| LD 5 | 2,830 | 22 | 32.89 | 0 | 0.0% | 100.0% |
| LD 5 | 3,400 | 22 | 32.89 | 0 | 0.0% | 100.0% |
| LD 5 | 3,960 | 22 | 32.89 | 0 | 0.0% | 100.0% |
| LD 5 | 4,530 | 22 | 32.89 | 0 | 0.0% | 100.0% |
| LD 5 | 5,100 | 22 | 32.89 | 0 | 0.0% | 100.0% |
| LD 5 | 850 | 26 | 21.35 | 5 | 0.2% | 99.8% |
| LD 5 | 1,130 | 26 | 21.35 | 0 | 0.0% | 100.0% |
| LD 5 | 1,560 | 26 | 21.35 | 0 | 0.0% | 100.0% |
| LD 5 | 2,270 | 26 | 21.35 | 0 | 0.0% | 100.0% |
| LD 5 | 2,830 | 26 | 21.35 | 0 | 0.0% | 100.0% |
| LD 5 | 3,400 | 26 | 21.35 | 0 | 0.0% | 100.0% |
| LD 5 | 3,960 | 26 | 21.35 | 0 | 0.0% | 100.0% |
| LD 5 | 4,530 | 26 | 21.35 | 0 | 0.0% | 100.0% |
| LD 5 | 5,100 | 26 | 21.35 | 0 | 0.0% | 100.0% |
